# Assessment of Occlusal Load Strength of Glass Ionomer Cement and Composite in Class V Cavities: An In-Vitro Study

**DOI:** 10.7759/cureus.49529

**Published:** 2023-11-27

**Authors:** Rakshitha V.S, Lakshmi Prabha J, Delphine Priscilla Antony S

**Affiliations:** 1 Department of Conservative Dentistry and Endodontics, Saveetha Dental College and Hospitals, Saveetha Institute of Medical and Technical Sciences, Saveetha University, Chennai, IND

**Keywords:** occlusal load strength, class v, universal testing machine, sensitivity, composite, glass ionomer cement

## Abstract

Background: Glass ionomer cement (GIC) is widely used in dentistry due to its chemical adhesion to dental tissues, biocompatibility, and anti-cariogenic potential but they have relatively weak mechanical properties. Resin composites have been widely regarded as the first choice for direct restorations but their polymerization shrinkage has remained a major problem. It has the potential to cause tooth debonding. The composite interface leads to postoperative sensitivity, secondary caries, enamel cracks, and microleakage. A restorative material's capacity to withstand occlusal stresses and support the remaining tooth structure depends on this property. Although class V restorations are predominantly done with GIC, this study was done to compare the strength of composite with the same. The GIC restore glass which is commonly used was tested against restofill composite. The main objective of conducting the study was to compare the compressive strength of the composite vs GIC in cervical cavities. So the aim of the study is to assess the occlusal load strength of GIC and composite in class V cavities using the universal testing machine.

Materials and methods: This study was employed as an in vitro study involving 20 natural central incisor teeth without any carious lesions. Class V cavity preparation was done and the selected teeth were divided into two groups of ten each. The cavities were filled with D Tech Restore GIC and composite restorations (restofill), respectively, polished, and then subjected to testing. An eccentric load was applied to the tooth structure using an Instron (Instron E3000 Electropuls, Instron, Norwood, United States) - Universal testing machine with a cross-head speed of 1mm per minute, and the stresses were further analyzed in the presence of an occlusal loading test using a stainless steel jig of 1mm diameter which led to the sectioning of the tooth buccolingually under the applied load.

Results: An independent t-test was used to assess the results, and it was concluded that the results were statistically significant (p<0.05) at p=0.034.

Conclusion: Conclusively, the results suggested that the occlusal load strength of the composite is greater when compared to GIC.

## Introduction

Class V lesions (G.V. Black's Classification) found on the buccal and lingual surfaces of the posterior teeth and the cervical third of anterior labially develop either through caries or abrasion or erosion of the tooth structure. Resin composite is commonly used to fill class V cavities in incisors, canines, and premolars; however, molars, are either filled with resin composite or glass ionomer cement (GIC). For its restoration, resin-modified glass ionomers can be utilized, but resin composite has better aesthetics [1]. Class V cavities are boxed in shape, have a floor, and four walls (occlusal, cervical, mesial, and distal), and a floor (pulpal). Retention grooves are positioned at the occluso-pulpal and cervico-pulpal line angles when amalgam is used [2].

Enamel and dentin are two distinct bonding substrates. Compared to enamel, dentin has a higher organic and water content but fewer minerals. Because of this, resin bonding to dentin is much more challenging and unpredictable than resin bonding to enamel. Diffusion of hydrophilic resin into and around the collagen fibers of the etched intertubular dentin produces a cohesive bond to dentin. Heavy occlusal loads are placed on teeth both during normal function and parafunction. The tooth may flex due to occlusal stresses, according to certain research [3]. Tensile and shear stresses are produced in the tooth's cervical region as it flexes [4]. This stress, which is produced in the cervical region, may deteriorate the margins of class V cavities or enhance microleakage [5]. The most reliable way to evaluate the quality of restorations is by conducting clinical trials. However, obtaining long-term clinical data takes too long, and during that period, the restorative materials used are frequently replaced. A restorative material's capacity to withstand occlusal stresses and support the remaining tooth structure depends on this property [6,7]. It was previously thought that class V restorations were only susceptible to forces from foods that stick to the surface and that they were not subjected to mechanical stresses distant from the area where occlusal pressures were exerted [7,8]. In the case of Mandibular teeth, high modulus macro-filled materials have been found to enhance the failure probability of class V restorations, particularly in patients who have strong occlusal loading as evidenced by the presence of wear facets [4].

In the 1960s, GIC was created as a result of an acid-base reaction between polycarboxylic acid and an essential fluoro-alumino-silicate glass powder in the presence of water [9,10]. Since then, the original formulation has undergone numerous modifications and enhancements. GICs that are conventionally set are hybrid materials that have both inorganic and organic components. These materials are constructed of tartaric acid-containing homo- and copolymers of acrylic acid and calcium fluoro-aluminium-silicate glass powder in aqueous solutions [11]. GIC has numerous applications in dentistry because of its unique properties [12,13]. It has anti-cariogenic properties, esthetics, the ability to release fluoride, low stimulation of the pulp, chemical adhesion to the tooth structure, microleakage at the tooth restoration interface, low cellular toxicity, and similar modulus of elasticity to the tooth structure and coefficient of thermal expansion [14-16] and it seems to be the most popular amalgam substitute for primary tooth restoration. GICs are widely used in medicine, primarily in reconstructive surgery and otologic, as well as orthopedics. Freshly prepared Ionomer-based material mixed with bone substitute is used during post-set hardening of self-curing bone substitute (Ionocem) developing a solid bond with the adjacent bone matrix. This cement doesn't generate any heat during the setting process; therefore, it won't heat up tissues or affect the heat-labile drugs that are part of the matrix phase of the cement [16-18]. Their primary medical applications include the stabilization of implanted devices, the resection or restoration of bone deformities, and the stabilization of bony fragments [19-21]. However, one of the most significant shortcomings of GIC is their susceptibility to moisture contamination, slow rate of setting, and dehydration during the early stages of setting it also has poor physical properties such as low flexural strength and low fracture resistance which prevents its use in high-stress bearing areas [22]. To enhance mechanical properties, Resin-modified GICs (RMGICs) were developed in the late 1980s [23,24]. These materials are more wear-resistant, moisture-resistant, more fracture toughness, and they last longer [22]. RMGIC has been introduced to overcome the shortcomings of GIC which has a higher initial strength than the original formulation [25].

Resin composites have been widely regarded as the first choice for direct restorations but their polymerization shrinkage has remained a major problem. It has the potential to cause tooth debonding. The composite interface leads to postoperative sensitivity, secondary caries, enamel cracks, and microleakage. The present generation of micro-filled composite resins are relatively resistant to wear, keep their high sheen, and are easily polished. Wettability and surface preparation experiments are typically followed by evaluations of bond strength [6]. For adequate isolation, Brinker rubber dam clamps are used for class V restorations along with gingival retraction. 

The main objective of conducting the study was to compare the compressive strength of the composite vs GIC in cervical cavities. Hence this study was aimed at assessing and comparing the occlusal load strength of composite and GIC in class V cavities using the Universal testing machine. 

## Materials and methods

**Figure 1:**
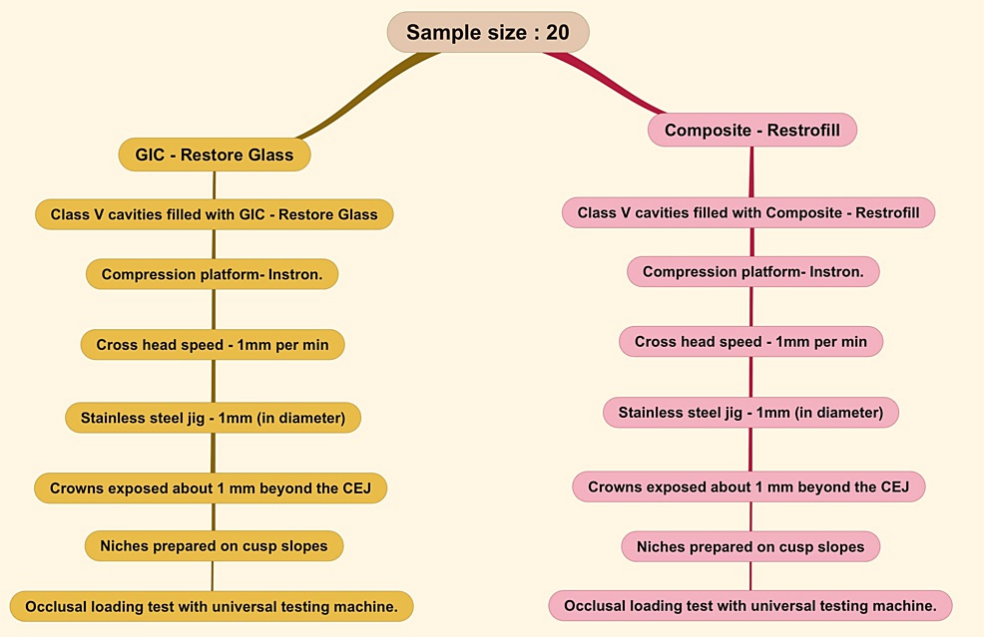
Methodology GIC: Glass ionomer cement; CEJ: Cementoenamel junction

The study has been approved by the Scientific Review Board with the reference number SRB/SDC/UG-2082/22/ENDO/140. This study was employed as an in vitro study involving 20 natural central incisor teeth without any carious lesions. Based on the article by Mazumdar et al., the power calculation was done and the sample size was calculated as 10 per group [26]. A standard class V cavity of 3mm mesiodistal width, 3mm occlusogingival height, and 1.5mm axial depth was prepared at the cementoenamel junction (CEJ) preparation was done using a slow-speed handpiece (Unicorn Dentmart Ltd., New Delhi, India) and a straight fissure bur (Neoendo SF-41, Orikam Healthcare India Private Ltd., Gurugram, India) with ideal depth. The selected teeth were divided into two groups of ten each and the cavities were filled with GIC (D Tech GIC restore glass) and composite restorations (Anabond composite restofill), respectively. The restorations were polished on the same day using a polishing bur and then subjected to testing. An eccentric load was applied to the tooth structure using an Instron (Instron E3000 Electropuls, Instron, Norwood, United States) - Universal testing machine with a cross-head speed of 1mm per minute and forces directed axially. The stresses were further analyzed in the presence of an occlusal loading test using a stainless steel jig of 1mm diameter which led to the sectioning of the tooth buccolingually under the applied load. These stresses were observed at the peripherals of the class V restoration when it was restored with GIC and with composite (Figure 1).

## Results

**Figure 2:**
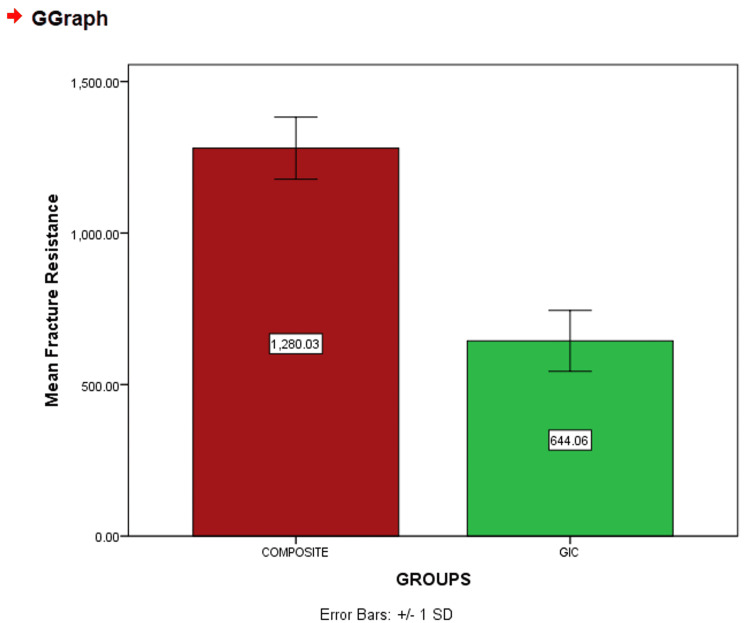
Graph showing mean fracture resistance between the two groups GIC: Glass ionomer cement

An independent t-test was used to assess the results, and it was concluded that the results were statistically significant (p<0.05) at p=0.034 as shown in the table below (Table 1). A graph was made based on the above results which shows the mean resistance between the two groups (Figure 2). As per the result of the study, it is inferred that the composite restorative material's capacity to withstand occlusal stresses, fracture resistance, compressive strength, and load-bearing capacity is more when compared to GIC. Except for the axial wall interface, the result was statistically significant (p<0.05), and a similar pattern was observed when composite was used as the occlusal restorative material.

**Table 1:** Mean fracture resistance between the two groups GIC: Glass ionomer cement

	Groups	N	Mean	Standard deviation	p-value
Fracture resistance	Composite	10	1280.0280	102.59739	0.034
	GIC	10	644.0640	100 46233	

## Discussion

Class V restorations are those placed on the gingival third of all teeth's buccal or lingual surfaces. Premature restoration loss due to cervical defects has been reported to be very common [7,27]. In the case of mandibular teeth, high modulus macro-filled materials have been found to enhance the failure probability of class V restorations, particularly in patients who have strong occlusal loading as evidenced by the presence of wear facets [4,6]. While chewing, teeth and restorations are always subjected to flexural and compressive forces. Furthermore, these stresses may be present to a greater extent during parafunction (e.g., bruxism and clenching) [28]. After years of follow-up on conventional composite restorations made indirectly or directly in high-stress-bearing areas, clinical studies have shown that one of the most frequent types of failure was fracture of the restoration. The longevity of the composite restorations was found to be more than 10 years which is relatively less when compared to GIC restorations. Similarly, in the current study, the occlusal load strength of the composite is greater than that of GIC [29,30].

Previously, class V restorations were thought to be only susceptible to the pulling pressures of sticky foods, with little thought given to the biomechanics of the tooth structure [31]. Gable was the first to speculate on the potential impact of occlusal forces on class V restorations. In addition, direct measurements of alterations in the occlusal-gingival diameter of class V preparations were done [3,32]. All indirect cavity preparations in the investigation showed lower fracture strengths, according to Mondelli [33]. Comparing the load to fracture of healthy human maxillary premolars with various cavity widths shows that the amount of tooth tissue removed has a relatively large impact on the load to fracture of teeth. The amount of tooth tissue lost was related to the change in the cervico-occlusal width of the cavity and the magnitude of deformation. Forces applied to a tooth's occlusal surface were found to cause stresses in a restoration located far away from the force's application point. The amount of tooth structure removed had an inverse relationship with fracture strength, with direct composite preparations being more resistant to occlusal load fracture than indirect preparations. They proposed that failure is caused by two different mechanisms. One of these is lateral excursive motions, which result in lateral cuspal motions and tensile stresses at the tooth restoration interface. The other is the heavy forces in centric occlusion that distort teeth vertically and create compressive and shear stresses at the interface of tooth restorations. Additionally, it has been demonstrated in vitro that the degree of coronal preparation is inversely correlated with cuspal flexure [34,35]. The presence of an occlusal restoration may negatively impact the preservation of a class V restoration if the tooth flexure theory is accurate. In comparison to an occlusal amalgam repair, the use of an adhesive composite has also been found to lessen cuspal flexure [36,37]. Additionally, it was noted that the interfacial tensions around class V composite and GIC restorations differed very slightly. When compared to composites, the GIC repair displayed significantly higher stresses at the gingival wall interface but marginally lower stresses at the occlusal enamel and dentinal wall. This might be explained by the fact that GIC is more brittle and has lower compressive, tensile, and flexural strengths than composites [38]. When amalgam served as the occlusal restorative material in a class V GIC restoration, the stresses at the class V interfaces were higher than they would have been in a scenario without the occlusal restoration. At the occlusal dentinal and gingival wall interface, the outcome was statistically significant. Therefore, it was observed that shear stresses at the occlusal enamel and dentinal contact significantly increased when occlusal amalgam was employed as a restorative material, especially when compared to class V restorations that had no occlusal restoration. It was suggested that as the composite restoration adheres to the tooth structure, the cusps' flexure decreases as the tooth structure is strengthened. In comparison to standard restorative composite, the results of this investigation demonstrated that glass fiber-reinforced dental composite resin had improved fracture toughness, compressive strength, and load-bearing capacity. It was discovered in the earlier investigation by Gaurav et al. that class V GIC caused greater strains at the gingival wall interface than composite [29]. Although none of the restorations were able to eliminate microleakage completely, it has been found that polymerization shrinkage and microleakage are lesser in composites when compared to GIC proving that composites are ideal for restorations. Thus in primary and permanent dentitions, resin-based composites can be utilized as a treatment option for class III and class V restorations [39].

The findings of a prior study [40], claim that in terms of fracture strength, the cavity depth is more important than the cavity width, and its findings disagree somewhat with those of the current study and other studies published elsewhere [41]. According to Cavel et al., more than 65% of the fractures in maxillary premolars were non-functional cusps fractures [42]. The limitations of the present study were, that only a smaller sample size was used and a single material was used against another. Variations in salivary pH and other practical difficulties occurring in vivo are not stimulated 100% in this study as it was conducted as an in vitro study. Further studies can be carried out to evaluate using RMGIC with composites.

## Conclusions

Within the constraints of this in vitro study, it has been found that the occlusal load strength of the composite is more significant when compared to GIC. Although class V restorations are predominantly done with GIC, this study was done to compare the strength of composite with the same. In this study, GIC restore glass which is commonly used was tested against restofill composite which shows that composite resins have better longevity when compared to GIC restorations. Although the retention rates in resin-modified GIC are higher compared to composites, it is seen that composite shows significantly higher bond strengths when compared to GIC. Hence this study can be improvised by comparison with other materials for a better understanding of the materials. Based on the findings and previous studies, composite can be recommended for wider applications such as in class V cavity restorations and for posterior teeth restorations as a core build-up material in stress-bearing areas. Variations in salivary pH and other practical difficulties occurring in vivo are not stimulated 100% in this study as it was conducted as an in vitro study. Further, for the future scope of this study, it can be performed in vivo with different materials along with proper follow-up and increased sample size.
